# Life Form and Life History Explain Variation in Population Processes in a Grassland Community Invaded by Exotic Plants and Mammals

**DOI:** 10.1371/journal.pone.0042906

**Published:** 2012-08-20

**Authors:** Lisa Castillo Nelis

**Affiliations:** Committee on Evolutionary Biology, University of Chicago, Chicago, Illinois, United States of America; University of Western Ontario, Canada

## Abstract

The existence of general characteristics of plant invasiveness is still debated. One reason we may not have found these characteristics is because we do not yet understand how processes underlying population dynamics contribute to community composition in invaded communities. Here I modify Ricker stock-recruitment models to parameterize processes important to community dynamics in an invaded grassland community: immigration, maximum intrinsic growth rate, self-regulation, and limitation by other species. I then used the parameterized models in a multi-species stochastic simulation to determine how processes affected long-term community dynamics. By parameterizing the models using the frequency of the 18 most common species in the grassland, I determined that life history and life form are stronger predictors of underlying processes than is native status. Immigration maintains exotic annual grasses and the dominant native perennial grass in the community. Growth rate maintains other perennial species. While the model mirrors the frequency of native species well, exotic species have lower observed than parameterized frequencies, suggesting that they are not reaching their potential frequency. These results, combined with results from past research, suggest that disturbance may be key to maintaining exotic species in the community. Here I showed that a continuous modified Ricker model fit discrete grassland frequency data well. This allowed me to model the dominant species in the community simultaneously and gain insight into the processes that determine community composition.

## Introduction

Whether general characteristics of plant invasiveness exist is a compelling theoretical question that has not been satisfactorily answered [1], [2], [3], [4]. Differences in some characteristics have been found between native and exotic species, but a current debate rages over whether these characteristics are truly determinants of invasiveness, or measures of exaptation to anthropomorphic changes [2], [3], [4]. One possible reason we have not found general characteristics for invasiveness is that we do not yet understand how underlying dynamical processes relate to population dynamics in invaded communities. Do native and exotic (*i.e.* non-native) species grow, spread, limit themselves or affect each other in some unidentified characteristic distinct from traits due to life history or life form? Do life history, life form, or native status groups differ in processes underlying population dynamics sufficiently to affect community composition?

To answer these questions, processes underlying population dynamics must be studied for multiple species within one community. Studying processes such as immigration, maximum intrinsic growth rate, density dependence, and limitation piecemeal in disparate communities a few species at a time could mask general trends. Instead, comprehensively studying these processes across a suite of native and exotic species within a single community would allow investigation of general patterns among native and exotic species while holding environment constant. If studied within a community, understanding the underlying processes driving dynamics can provide general knowledge about how these processes directly relate to community composition. Comparative community analyses have generally focused on phenotypic traits [5], but the processes of immigration, intrinsic growth rate, density-dependence, and species interactions are more directly linked to differences in species success and population dynamics.

More generally, exploring processes underlying population dynamics in a community can clarify whether such differences within and between native status groups (*i.e.*, native versus exotic) are real, or are reflections of processes that differ between life history (*i.e.*, annual versus perennial) or life form (*i.e.*, grass versus forb) groups. For example, annual species tend to be both early successional colonizers and common exotic plants [6]. Are differences commonly found between native and exotic plants due to native status, or simply because annuals are better at quickly grabbing resources in disturbed areas? In a community with a mix of native status life history and life form groups, we can separate native status from other plant characteristics.

An excellent community for this study is the grasslands in Vaquería Valley, Robinson Crusoe Island, Chile. In this multiple-origin community, exotic species make up the bulk of the diversity (47 of 56 species), although one native bunch grass, *Nassella laevissima*, makes up the majority of the biomass. A non-native herbivore, European rabbit (*Oryctolagus cuniculus*), and non-natural soil disturbances may also be influencing processes underlying population dynamics differentially between native and exotic species. This system calls to mind specific questions: how are native species maintained when so overwhelmed with invasive species? Do immigration, maximum intrinsic growth rate, self-regulation (*i.e.* limitation of conspecifics though density dependent processes) and limitation by other species (limitation of heterospecifics though competition or allelopathy) differ between the native and exotic species? Do these processes differ due to the presence of rabbits or soil disturbance?

To answer these questions, I describe key processes underlying population dynamics for the 18 dominant grassland plant species in Vaquería valley. I parameterize immigration, maximum intrinsic growth rate (hereafter growth rate), self-regulation, and resistance (*i.e.* limitation by other species) using Ricker models to model plant frequency data [7]. I then conduct a comparative analysis testing whether processes differ systematically among members of the community with different life histories, life forms, and native status. I determine whether herbivore presence or disturbance changes processes. To clarify how changes in these processes affect long-term population dynamics within a shared community I also simulate populations of species in different treatments in a multi-species stochastic simulation. To hold the potential confounding effects of environmental variation constant, I sampled all species in the same grassland community on Robinson Crusoe Island over the same time period and simulated them in a stochastic multi-species Ricker model. Specifically, I hypothesize that:

1. Immigration, growth rate, self-regulation, and limitation by other species vary predictably between annuals and perennials and between grasses and forbs due to differences of structure and function in these groups. For example, early successional species could be expected to colonize quickly but lose in competition. Therefore, annuals and grasses may have higher immigration and stronger limitation by other species because they tend to be early successional species.
2. Plant population dynamic parameters vary predictably between native and exotic species. For example, native species accustomed to the abiotic conditions may have higher growth rates and lower self-regulation.
3. Parameters vary predictably due to herbivory and disturbance. For example, unpalatable species may have higher growth rates under grazing pressure.

It is particularly interesting to determine whether native status significantly predicts variation in processes independent of life history or life form groups. If so, it would suggest that native status confers some unknown group characteristic in addition to life form and life history predictive of processes underlying population dynamics.

## Materials and Methods

### Study System

This study took place in an invaded grassland in Vaquería Valley on Robinson Crusoe Island, Juan Fernández Archipelago, Chile. The grassland does not appear to have an invasion front; plant species occurred over large portions of the valley. European rabbits, an exotic selective generalist herbivore, also occur throughout the grassland. European rabbits were released in 1935 [8]. As Robinson Crusoe Island has no native mammals, amphibians, or reptiles, rabbits are a novel source of herbivory and disturbance. In fact there were probably few regular disturbances before human discovery in 1574, as there were no large herbivores [9], and infrequent fire [10]. Introduced rats, mice, rabbits, goats, horses and cattle were present in Vaquería until the larger hoofed mammals were fenced out in 1987 (Leiva personal communication 2010). Unfenced grasslands with similar species composition occur in many other places on the island ([11]), though some areas are overgrazed by cattle and horses (personal observation 2004–2007).

Vaquería Valley is within the Parque Nacional Archipiélago Juan Fernández (a Chilean National Park). All necessary permits were obtained for the described field studies from the Corporación Nacional Forestal de Chile (CONAF), Región V.

### Experimental Treatment Application

To test effects of rabbit presence and disturbance on plant species frequency, a team of assistants and I constructed eight experimental rabbit exclusion blocks in 2004. The blocks measured 20 meters by 40 meters and were spaced throughout the valley (Fig. S1). Each block was split into two 20×20 meter halves. One half was randomly selected and fenced to exclude European rabbits, and the other half was installed with fence posts and disturbed around the perimeter to mimic fence installation, but was not fenced.

Within each half-block were 48 permanent subplots (0.5 meter×0.5 meter). In 2004, we disturbed half of the subplots once, by manually turning over the soil as in a garden. Rabbit and rodent digging, human disturbance, and erosion resulting from previous disturbances are causes of soil disturbance in this system. Since these disturbances are not native and could have long-term implications on the plant community, I implemented the disturbance treatment to gain insight into plant community recruitment and recovery under a non-native disturbance regime. The treatments of rabbit presence or absence crossed with soil disturbance resulted in four treatments.

Teams of field assistants and I censused all subplots once each year, in austral spring, from 2004 through 2007. We used quadrats divided into twenty-five 10 by 10 cm quadrat squares. Within each of the 25 quadrat squares, all plants were identified to species and their presence was recorded. Presence data were collected in each quadrat square instead of count or plant measurement data for two reasons: 1) I was able to track species frequency for all species encountered, and 2) time efficiency allowed us to sample a higher percentage of the grassland and capture sufficient plant diversity within each treatment to saturate species abundance curves [12], [13] (Fig. S2). In this paper, frequency for a given species is the number of quadrat squares within a subplot in which that species was present, ranging from 0 to 25 (see [4] for more information on data collection methods). While these frequency data are discrete, here I ignore the discreteness and model them as continuous for simplicity.

### Model Construction, Parameterization and Selection

I chose to use Ricker models [14] to characterize the population dynamics in my plant system primarily because they fit my data well. In an initial examination of data, I graphed frequency at time zero (stock) versus frequency at time one (recruits) for each species. Most of the 18 species showed Ricker-like curves (Fig. S3). In a posterior examination of the data, I graphed the parameterized equations with the data and again found an excellent fit (Fig. S4). I also chose Ricker models because they are simple and well understood models that offer a generalized framework of key population parameters that can exhibit a range of dynamics [15], [16], [17], [18]. I here focus on modifying the Ricker model to include important aspects of my biological system to compare processes between species and groups. I examine the following processes underlying population dynamics: immigration, maximum intrinsic growth rate, self-regulation, and resistance by other species of plants. Exploring many different models is beyond the scope of the current paper.

To my knowledge, Ricker models have not previously been fit to plant community dynamics. Thus, expanding the use of Ricker models to plant dynamics will provide a new method of population analysis, complementary to direct measures of plant demographics. Fitting Ricker or similar models can help researchers to understand key underlying processes (e.g. growth rate, self-regulation, etc.) in systems where it is difficult to collect direct measures of plant population dynamics. Also, by modeling instead of collecting direct measures, it is possible to collect data for many species at once, which will encourage studies of demographics in a community context. The following equations were parameterized using frequency data [7] collected over three years, 2005 through 2007. Frequency data were used because they allowed me to implicitly include space in the Ricker model. Spreading in space is necessary for invasion; thus, parameterizing by frequency in space gives us a better picture of local plant species expansion.

The basic Ricker model [14] is: 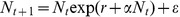
(1)where *r* is population maximum intrinsic growth rate and *N_t_* is the censused population at time *t*. A negative *α* indicates self-regulation; a positive *α* indicates self-facilitation. Since, in this study, *α* is almost always negative, I will refer to *α* as self-regulation. Growth rate parameterized from frequency is actually the increase in spatial segments occupied by the species on a local scale. Self-regulation is the restriction of spatial spread by conspecifics. The error term in this and subsequent equations is normally distributed noise because I expect that it is the result of many random factors that are independent of the parameterized processes.

In addition to growth rate and self-regulation, conspecific seed immigration is an important interaction in this system. Immigration is the increase in spatial occupancy not accounted for by previous residents (stock). I modeled immigration by adding an immigration term *I* to *N_t_*:
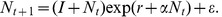
(2)


Thus, the model reflects our sampling during the cycle of the natural system, in which seed input occurs first, followed by competition, followed by the annual census when the plants are in seed.

Another potentially important component of a grassland community is limitation by other species of neighboring plants, which can act to resist spread. To include limitation by other species I tested two different resistance terms by further expanding the Ricker model as follows:
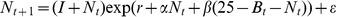
(3)

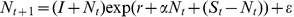
(4)where 25 is the highest frequency any one species can reach, *B_t_* is the count of space with no plants at all (*i.e.*, bare space), and *S_t_* is the sum of the counts of all species in the same sampled area from which we censused *N_t_*. A negative *β* indicates resistance by other species; a positive *β* indicates facilitation by other species. In this study, *β* is almost always negative and is therefore referred to as community resistance. Resistance is the restriction of spatial spread by other species. In Eq. 3, I suggest that the most important aspect of interaction with other species are those spaces in a plant's neighborhood that do not contain conspecifics of the plant species but are of high enough quality to contain other plant species. Many areas of bare space are unsuitable for plant growth, such as those covered by a rock or a dense mat of dead vegetation. Therefore, bare space and spaces occupied by conspecifics are subtracted from the 25 total quadrat squares to leave the number available for colonization. In contrast, in Eq. 4, I suggest that the most important aspect of interaction with other species is the total estimated frequency of other plants (0–25 possible for each of the 57 species) in the quadrat. It should be noted that all species contribute to *S_t_* and *B_t_* in proportion to their abundance, although each species experiences the effect of density dependence in different intensities. In many situations, impacts of individual species are included in such models (e.g., [19]). I chose not to estimate species-specific interspecific effects because sample sizes were insufficient to do so for the many additional parameters required.

Using Matlab (Release 2009b), the models were parameterized for the 18 species with population abundances high enough to accurately parameterize the model (Table 1). Four of the 18 species were native to Robinson Crusoe Island; the other 14 were exotic species from Europe, the Mediterranean, and South America. For each species, the models were parameterized for species pooled across treatments, and for each of the four rabbit crossed with disturbance treatments individually. Pooled treatments were parameterized across all of the 768 subplots regardless of treatments that were assigned to each subplot. Models fit by treatment were parameterized on the 192 subplots corresponding to each treatment. The likelihoods of these model fits were summed for comparison to pooled fits. The log-likelihood equation was
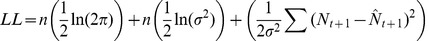
(5)where n is the sample size, *σ^2^* is the variance, and *N_t+1_* is the observed population.

**Table 1:** The 18 plant species modeled in this study.

Species Number	Species	Native status	Life form	Life history
1	*Avena barbata*	exotic	grass	annual
2	*Aira caryophyllea*	exotic	grass	annual
3	*Anthoxanthum odoratum*	exotic	grass	perennial
4	*Briza maxima*	exotic	grass	annual
5	*Bromus hordaceus*	exotic	grass	annual
6	*Bromus stamineus*	exotic	grass	perennial
7	*Briza minor*	exotic	grass	annual
8	*Dipsacus sativus*	exotic	forb	annual
9	*Hypochaeris glabra*	exotic	forb	annual
10	*Hypochaeris radicata*	exotic	forb	perennial
11	*Juncus imbricatus*	native	grass	perennial
12	*Nassella laevissima*	native	grass	perennial
13	*Nassella neesiana*	native	grass	perennial
14	*Piptochaetium bicolor*	native	grass	perennial
15	*Rumex acetosella*	exotic	forb	perennial
16	*Sonchus asper*	exotic	forb	annual
17	*Sonchus oleraceus*	exotic	forb	annual
18	*Vulpia bromoides*	exotic	grass	annual

After parameterizing each of the four models for both pooled and treatment data, I calculated the AIC's for each of the eight fits (four models x pooled and treatment; [20]). I then compared AIC values to find the best fit for the data. To test whether groups of plants fit certain models better, I conducted a MANOVA and an ANOVA with model number as the dependent variable. Independent variables were life history as an annual or perennial, life form as a forb or a grass, and native status as independent variables.

### Population Dynamic Parameters

To test whether population dynamic parameters varied systematically across ecological groups, I conducted a MANOVA across all best-fit parameters, using species as replicates (JMP 9). To clarify differential responses among parameters, I conducted mixed effects general linear models (GLMM) for each parameter using species as replicates (JMP 9). For both analyses, the dependent variables were the four best-fit parameters or transformations of the best-fit parameters: immigration, growth rate, self-regulation, and resistance. Immigration, growth rate, and self-regulation had non-normal distributions and were transformed to fit the assumption of normality of the statistical model. Each parameter was transformed with different transformations because each had different initial distributions. To select the transformations, I first added 0.0001 to immigration (*I*) and self-regulation (α) to eliminate zero values so transformations would work. I then examined the likelihood of normal fits between the distributions of the original data and several standard transformations. The transformations with the highest likelihoods and strong overlap between the mean and the median were selected. Immigration (*I*) was log*_e_*(*I*+0.0001) transformed. Growth rate (*r*) was √*r* transformed. Self-regulation (α) was 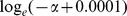 transformed.

Independent variables in the MANOVA and GLMM were the main effects of life history, life form, native status, rabbit, and disturbance. All possible interactions were tested but were insignificant, and so were removed from the model. Species of plant was included in the GLMM as a random independent effect to account for the variation caused by idiosyncratic species differences.

### Simulation of Populations

I simulated the best-fit model to explore its dynamic implications using a multi-species stochastic simulation for 2000 time steps. The stochastic error was added as a last term to the model in the simulation. The error was the product of the variance of the residual sum of squares from the model fit and a random number from a normal distribution. The stochastic model allowed some species to persist that went extinct in the deterministic model, and kept the simulated rank-abundance distribution more similar to the real rank-abundance distribution (Figs. S7 and S8). The stochastic component had a magnitude of mean 0.091 and a standard deviation of 4.145.

The model is multi-species because each of the 18 species was calculated at each time step, a new sum of all species was calculated (*S_t_*), and the new value *S_t_* was incorporated into the next iteration. Simulated frequency was bounded from zero to 25 to reflect the data, although few species reached such frequencies often. Bare space (*B_t_*), which was a parameter in equation 3, was not used in the simulation because it was not a parameter in the best-fit model.

The initial conditions of the simulation did not alter mean frequencies of simulated populations. To determine the importance of initial conditions, the simulation using the best-fit model was run twice, once with the initial conditions set to zero, and once with initial conditions set to the real population means from the data. I found that the mean simulated frequencies for each treatment and each species were not significantly different (*p* = 0.2056) by using a matched pair t-test (R version 2.11). For simplicity, results presented here are from the simulation with initial conditions set to zero.

From the simulation results, I calculated the temporal coefficients of variation, number of times the simulated population went locally extinct, mean population frequency, and maximum population frequency reached. These characteristics were calculated after omitting the first 100 time steps to eliminate transient fluctuations.

To determine how groups or treatments affected simulated characteristics, I conducted GLMMs using characteristics of the simulated model dynamics (or transformations) as dependent variables. Dependent variables were the coefficient of variation squared, the square root of the number of times each species went to local extinction, maximum population frequency squared, and the log transformed mean population frequency. Independent fixed effects were life history, life form, native status, rabbit, disturbance, and all possible second-degree interactions. Species of plant was included as a random effect. Finally, I calculated correlations among untransformed simulated population characteristics to determine if there were unusually strong correlations that might suggest plant survival strategies.

## Results

The best fitting model overall was Eq. 4 fit by treatment (see parameters, AICs, and R^2^s in Table S1 and S2). Model 4 included immigration (*I*), maximum intrinsic growth rate (*r*), self-regulation (α), limitation by other species (*β*), and total numbers of other species (*S_t_*). For 12 of the 18 plant species, Eq. 4 fit by treatment had the lowest or indistinguishably low AIC scores among the eight fits. For all but two species, models with treatment-specific parameters described the data better than those with treatment-independent parameters. Neither life history, life form, nor native status predicted which equations or parameters fit best (General Linear Model *p*>0.05). The best fitting model, Eq. 4 fit by treatment, fit the data well. The mean R^2^ for model fits was 0.433, and the median was 0.522 (Table S1, Fig. S4).

### Population Dynamic Parameters

**Figure 1:**
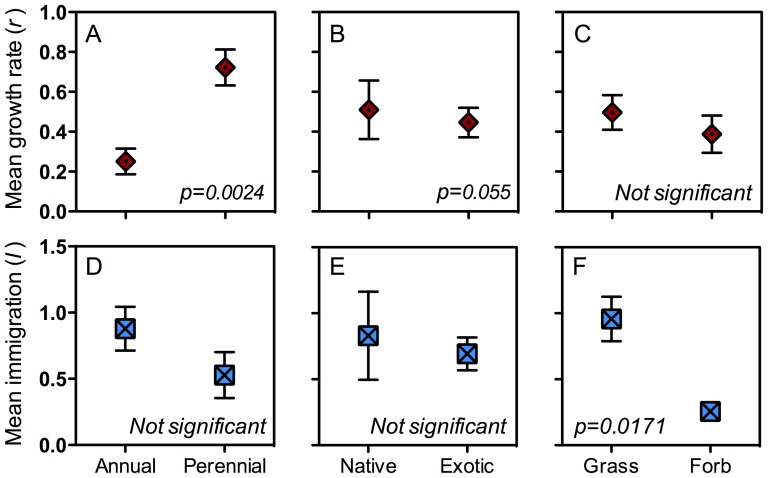
Immigration (*I*) and maximum intrinsic growth rate (*r*) by life history, native status, and life form. A, B, &C) Mean growth rate, and D, E, & F) immigration for life history, native status, and life form. Error bars are standard error among treatments and species.

**Figure 2:**
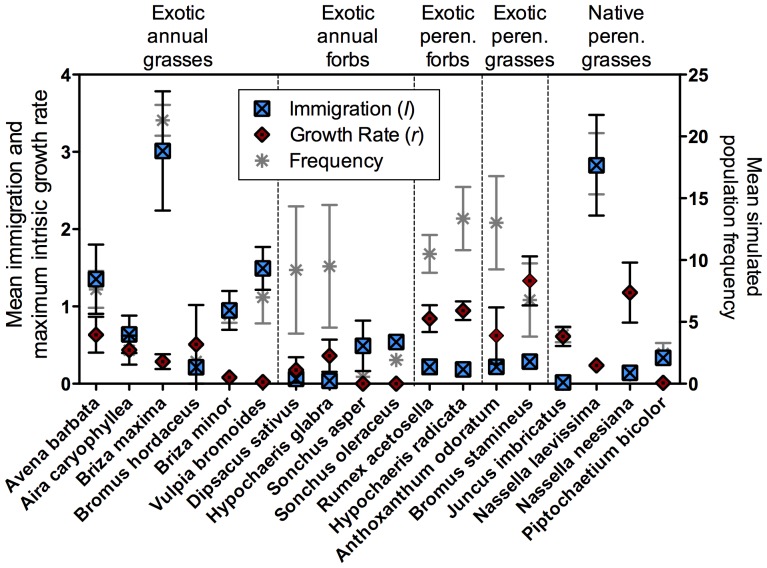
Immigration (*I*), maximum intrinsic growth rate (*r*), and mean simulated frequency by species. The left y-axis shows immigration (squares) and growth rate (diamonds), and the right axis shows simulated population frequency (asterisks). Error bars are standard error among treatments. Please note that while both immigration and growth rate are on the same axis, they cannot be directly compared to one another.

Significant differences were found among population dynamic parameters for life history, life form, and native status groups (whole model Wilks' Lambda, F(20, 209.9) = 2.237, *p*<0.0001), but not for rabbit or disturbance treatments (Tables S3 and S4). The follow-up general linear mixed-effects model (GLMM) showed that of the fixed main effects, grass species had significantly higher immigration than did forbs (GLMM *p* = 0.017), and perennial species had significantly higher maximum intrinsic growth rates than did annuals (GLMM *p* = 0.002, Figs. 1, 2, and S5). Native species had marginally significantly higher growth rates than did exotic species (GLMM *p* = 0.055, Fig. 1), but this is largely due to *Nassella neesiana*, one of the four native species, having very high growth rates (Fig. 2). Neither self-regulation nor resistance by other species varied significantly by group (Table Fig. S10).

### Simulation of Populations

Equation 4 fit by treatments was the best-fit model and was used to simulate all species. The simulated communities have higher species richness and frequencies than was observed from parameterized data, but the simulated communities still mirrored patterns found in the observed data (Figs. S5 and S6). For example, species richness and Shannon index scores were fairly uniform across treatments in both simulated and observed data, and evenness tended to be higher in treatments without rabbits in both observed and simulated data (Fig. S6). Species with higher frequencies in observed data also tended to have high frequencies in simulated data, although observed and simulated frequencies were closer for native than exotic species.

Simulating the parameterized models allowed me to examine long-term characteristics of the community. Significant differences were found among simulated population characteristics for life history, life form, and native status groups and for the rabbit exclusion treatment (whole model Wilks' Lambda, F(20, 209.9) = 3.3009, *p*<0.0001), but not for the disturbance treatment (Tables S5 and S6).

**Figure 3:**
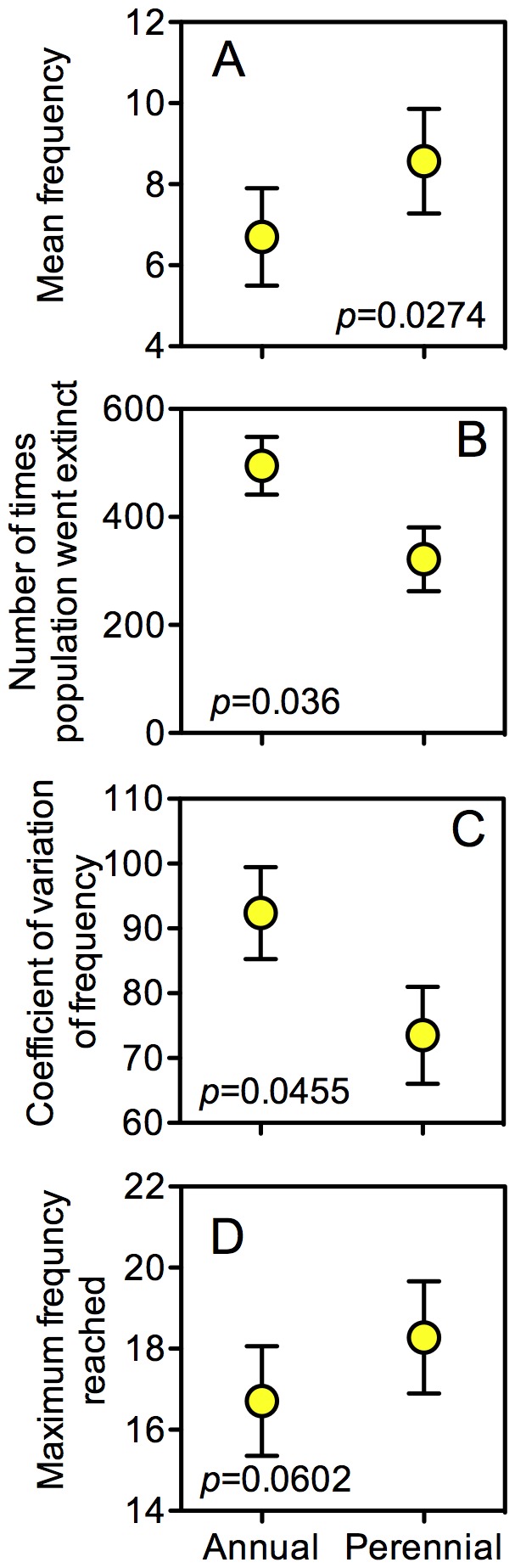
Characteristics of simulated populations for annual and perennial species. Characteristics of the simulated populations separated by life form for A) mean frequency, B) number of times population went extinct, C) the coefficient of variation, and D) the maximum frequency reached. Error bars are standard error among treatments and species.

**Figure 4:**
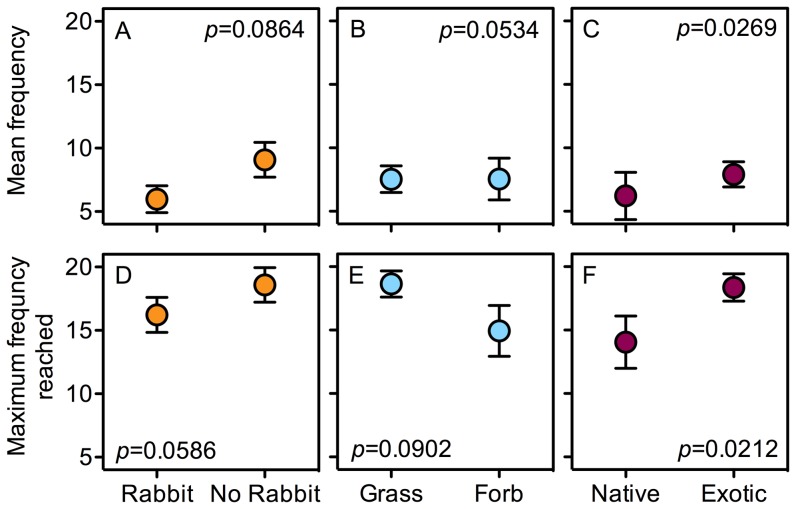
Mean and maximum frequency for rabbit treatments, life form, and native status. A, B, & C) Mean frequency, and D, E, & F) maximum frequency reached for rabbit treatments, life form, and native status. Error bars are standard error among treatments and species.

In simulations, populations varied most strongly by life history; annual species did not seem as well adapted to the environment as perennial species (Fig. 3). The GLMM of simulated populations showed that annual species remain at lower mean frequencies (GLMM *p* = 0.0274), go extinct more often (GLMM *p* = 0.036), have higher coefficients of variation (GLMM *p* = 0.046), and remain at lower maximum frequencies (GLMM *p* = 0.0602) than perennials. Populations also marginally significantly varied in mean and maximum frequency due to rabbit exclusion and life form (Fig. 4). Rabbit exclusion increased both the mean and the maximum frequency of all plant species (GLMM *p* = 0.0864 and *p* = 0.0586, respectively), most likely as a result of reduced grazing and disturbance. While the difference in frequency caused by rabbits may seem small, it is biologically meaningful. For example, mean frequency changed from 5.98 with rabbits to 9.08 without rabbits, a 52% increase in number of quadrat squares occupied. In contrast, while grass species reached higher mean and maximum frequencies than did forbs (GLMM *p* = 0.0902, *p* = 0.0534, respectively), the increase in mean frequency from 7.52 to 7.54 is unlikely a biologically meaningful change.

Simulated populations of exotic species reached significantly higher mean and maximum frequencies than did native species (GLMM *p* = 0.0269 and *p* = 0.0212, respectively), but whether or not this is biologically important is debatable (Fig. 4). There are only four native species which were parameterizable, three of which had relatively low frequencies, and one of which is the dominant species in the community and which maintained high observed and simulated frequencies (Fig. 2).

All dynamic characteristics of the simulations were correlated (correlation coefficients >|0.7|), which is unsurprising as they were not independent. However, the coefficient of variation was strongly positively correlated with the number of times the simulated population went extinct (correlation coefficient  = 0.979), suggesting that strategies that focus on reducing variability may be important in facilitating maintenance of species [21].

## Discussion

This study demonstrated that a modified continuous Ricker model fits discrete grassland frequency data well. The strong fit of the model allowed me to determine that in this community there does not appear to be a characteristic of invasiveness separate from plant life-history traits. It also clarified processes that determine community composition. In this grassland, immigration and growth rate seem the most important processes for maintaining species in communities, and perennial species appear to be better adapted to this study system than are annuals.

Finding a characteristic of invasiveness that would allow us to predict which plant species will become invasive is theoretically compelling. Unfortunately, my study did not identify such a characteristic. Instead, it seems that the plant traits of life history and life form best explain variation in the processes underlying population dynamics. Specifically, life history and life form can predict the growth rate and immigration of plant populations (Figs. 1, 2, S9). Perennial species have significantly higher growth rates than annual species, suggesting that they invade new areas by spreading locally through seed production and or through vegetative growth. Grass species have significantly higher immigration rates than forbs, which means they probably disperse their seeds more widely.

Perennial and grass species most likely have higher growth rates and immigration because they are exapted for harsh climatic conditions. In Vaquería Valley plants must tolerate cold wet winters (rarely as low as 0°C), hot dry summers, soil less than a meter deep directly above bedrock, and soil lacking in nutrients [22]. Grime (2001) found perennial species are stress-tolerant to extended dry conditions such as summers in Vaquería, and thin-bladed grasses are one of the most common plant types in areas with poor soils [23]. Native plants, which are all perennial graminoids, should have both the advantage of high growth rate and high immigration. However, individual native plant species vary in how they exploit these advantages (Figs. 2 and S9).

For some species, parameterizing processes confirmed my intuition of which processes maintained them in the community. For example, *Nassella laevissima*, the dominant native bunch grass, had an effective method of seed dispersal that appeared to be important to its dominance. Each long and thin grass blade bearing a spikelet of small seeds breaks off at the base and is carried into the air in swirling golden clouds. These blades of grass are then distributed widely across the valley (Nelis pers. obs. 2004–2011). In confirmation that dispersal is important to *N. laevissima*'s dominance, the Ricker model found that it has an extremely high immigration rate and relatively low growth rate. *Nassella neesiana* and *Juncus imbricatus*, rarer native species, appear to utilize growth rate over immigration for their persistence. *N. neesiana* has large seeds that are unlikely to fly very far, grows in large clumps of individuals, and is patchily distributed throughout the valley. *J. imbricatus* grows vegetatively and has very low flowers that probably do not disperse far. These natural history observations are supported by the model results, they both have relatively high growth rates and low immigration rates. For other species, parameterized processes provided a basis for forming hypotheses for which I had no intuition. For example, the native species *Piptochaetium bicolor* is rare, so I did not have enough natural history observations to hypothesize which process maintain it in the community. This model showed that although both immigration and growth rate of *P. bicolor* are low, immigration is relatively higher and may be the more important of the two processes.

One limitation of modeling with frequency, is that abundance does not always correlate with occupancy [24]. For example, the model results for *Briza maxima* do not correlate directly with its observed dominance in the field. In the model, *B. maxima* has a higher growth rate, higher immigration, less limitation by other species, and in the simulation is more frequent than the dominant observed species, *N. laevissima*. Why therefore, has it not surpassed *N. laevissima* to become the dominant species in either frequency or percent cover in the field? In established *N. laevissima* grassland, tufts of the native bunchgrass are surrounded by a matrix of dry dead organic matter limiting sprouting of other species. *B. maxima* individuals can grow on top of *N. laevissima* tufts or directly on the organic matter, but individuals are small and do not produce many seeds (Nelis pers. obs. 2004–2010). In the field, *B. maxima* is strongly benefited by disturbance [25], and appears to grow to large size and produce many seeds only when in disturbed areas.

The dependence of exotic species on disturbance probably explains the difference between simulated and observed frequencies among exotic species as well (Fig. S5). The simulation mirrors the observed frequency of native species well, but predicts that most exotic species should have higher observed frequencies than they do. Past research has shown that exotic species are facilitated by disturbance [25]. The current research suggests that the mechanism for facilitation is different for exotic annual and perennial species. Assuming exotic annual species depend on immigration to persist in the community, soil disturbances may be critical in opening areas to immigrate into. In contrast, exotic perennial species spread locally, so may need disturbance to facilitate a foothold in the community. Non-native soil disturbances such as exotic mammalian digging, human perturbation, and erosion from past disturbances appear to be supporting these populations of exotic species. If soil disturbance could be eliminated, many of these species could potentially become locally extinct.

Data suggests that exotic species are dependent on disturbance, but in this study there was no response to disturbance in either the parameters or in simulations. This is probably because the current study did not reflect the initial advantage of disturbance. The models were parameterized with data starting the year after disturbances were colonized to eliminate variation resulting from slightly elevated misidentifications of plants in the first year of the study. This means that the initial jump from no species to exotic species [26] was excluded from the model, and exotic species were not directly shown to have an advantage from disturbance.

While native persistence through local adaptation and exotic persistence through soil disturbance is supported by this study, more research is necessary before these hypotheses can be deemed the definitive explanations. The native ranges of most of the exotic species in this system are defined coarsely by continent, making it hard to determine to what environmental conditions they are adapted. Also, there is little data on dates, numbers of founders, or origin of plant introductions. Some species, such as *Avena barbata*, probably invaded over 200 years ago [9], while other introductions are assuredly more recent. Thus, the impact of rapid evolution on adaptation of exotic species to local environmental conditions cannot be resolved and is an aspect that clearly warrants further investigation. Also, there is little research on native and exotic arthropods in the system. While I have not seen damage from invertebrate herbivory, it could still be cryptically occurring and differentially affecting native and exotic plant recruitment or survival.

Neither immigration, growth rate, self-restriction, nor community resistance significantly differed with the exclusion of European rabbits or with disturbance. But the exclusion of rabbits did result in biologically significant differences in population frequency. Excluding rabbits increased both the mean and the maximum frequency plant species reached, suggesting that rabbit herbivory and or disturbance reduce plant frequency. Slight variation in dynamic parameters can apparently cause important changes in population frequency, making their study even more important.

In summary, continuous Ricker models fit discrete frequency data well, which allows insight into processes underlying population dynamics and determining community composition. By modeling and simulating the dominant species within one community together, I clarified which processes are important for maintaining species and groups in the community. In Vaquería Valley, life history and life form are strong predictors of immigration and maximum intrinsic growth rate, which seem to be the most important processes for maintaining species in the community. I did not find a characteristic of invasiveness separate from plant life history traits, but I did determine that native species in this community are well adapted to local environmental conditions, and that exotic species are likely maintained partially through disturbance. Understanding these differences clarified mechanism maintaining native and exotic species in the system, and could be informative in other systems as well.

## Supporting Information

Figure S1 **Experimental rabbit exclusion and disturbance blocks.** A) Diagram of experimental blocks. Solid lines indicate fencing, dashed lines indicate fence posts but no fence. Filled squares are disturbed subplots, empty squares are undisturbed subplots. B) A map showing location of all eight blocks overlaid onto a satellite photo of Vaquería valley. The blue line is the stream. For scale, the small dark dot to the left of the stream near the beach is the cabin were researchers stay when in the valley. C) A photo of block 6 in 2005. To the left is outside of the fenced area, to the right is inside. D) This picture shows a subplot with a portable quadrat on it for collecting presence data.
(TIF)

Figure S2 **Species abundance curve.** This species abundance curve shows the accumulation of species in the no rabbit not disturbed treatment across all experimental blocks in 2009. Each treatment has 196 subplots and 4800 quadrat squares, the 10 cm×10 cm samples shown on the x-axis. The curve is fairly flat well before all samples within one treatment are collected. This is strong evidence to support that sampling is sufficient to captures the diversity present within each treatment. The calculation for this figure was done using EstimateS (http://purl.oclc.org/estimates).
(EPS)

Figure S3 ***N_t_*** **versus** ***N_t+1_*** **for each species 2005–2007.** For many of the species (3, 5, 6, 9, 10, 13, 14, 16, and 17) the curve is a textbook Ricker curve. For other species (1, 8, and 15) the curvature of the line resembles a Ricker curve that does not descend. The remaining species (2, 4, 7, 11, 12, and 18) do not resemble Ricker curves. However, some of the species that do not resemble Ricker curves fit the model exceptionally well. For example, species 11 (*Juncus imbricatus*) has an R^2^ of 0.916.
(TIF)

Figure S4 **Model fit with data.** The model fit for model 4 for four species in the no rabbit no disturbance treatment are shown. A) *Juncus imbricatus* (species 11), has the best fit with an R2 of 0.916. B) *Sonchus asper* (species 16) has the worst fit with an R^2^ of −0.040, this appears to be due to a lack of data. C) *Nassella laevissima* (species 12) is the dominant bunch grass and fits the model well with an R^2^ of 0.511. D) *Briza maxima* (species 4) is the dominant exotic species and fits the model well with an R^2^ of 0.400.
(TIF)

Figure S5 **Simulated and observed frequency.** Mean simulated frequency (asterisks) and observed frequency (circles) for 2005–2007, from which the model was parameterized. The error bars are standard error among treatments. Error bars on the observed data are too small to see. The simulated data overestimate the frequency of almost all of the exotic species, but in general, the trend in the frequency is the same. Frequent observed species are generally frequent modeled species.
(EPS)

Figure S6 **Simulated and observed diversity.** A) Species richness, B) Shannon-Wiener, and C) Evenness of both simulated (diamonds) and observed (squares) populations. Because the simulation overestimates the frequency of each species, it also overestimates the species richness and the Shannon-Wiener diversity index. However, the pattern of similar richness and diversity among treatments remains, and the pattern of less evenness in plots with rabbits holds between both observed and simulated data. Error bars, which are too small to see, are standard error.
(EPS)

Figure S7 **Rank-frequency, mean frequency, immigration, and growth rate of simulated data.** Rank-abundance style graphs with A) mean simulated population frequency, and B) rank-frequency of stochastic model. C) mean simulated frequency, and D) rank-frequency of deterministically simulated model. Immigration (diamonds) and growth rate (squares with an x inside) are shown on the right axis.
(TIF)

Figure S8 **Rank-frequency, mean frequency, immigration, and growth rate of simulated data.** Rank-abundance style graphs with A) mean observed population frequency 2005–2007, and B) rank-frequency of observed data 2005–2007. C) mean observed frequency 2009–2010, and D) rank-frequency of observed data 2009–2010. Immigration (diamonds) and growth rate (squares with an x inside) are shown on the right axis.
(TIF)

Figure S9 **Immigration (** ***I*** **) and maximum intrinsic growth rate (** ***r*** **) for each species.** Immigration (squares) is on the left axis, and growth rate (diamonds) is on the right axis. This graph has both parameters on their own axis to clarify their relative distributions.
(EPS)

Figure S10 **Self-regulation (** ***α*** **) and community resistance (** ***β*** **) for each species.** Community resistance (beta) is shown with squares on the left axis, and self-regulation (alpha) are the stars on the right axis.
(EPS)

Table S1 AIC and R^2^ fit. Columns two through nine show the AICs for each equation and treatment fit. Column ten shows the fit (numbered 1–8 in parentheses in column titles) that had the lowest AIC. The difference between the lowest AIC and the overall best fit equation, Equation 4 by Treatment (fit 8), is shown in the penultimate column. The final column shows the R^2^ for the fit between Eq. 4 by Treatment to the data for each species. As you can see, most of the model fits that have an AIC lower than that of Eq. 4 by Treatment still fit Eq. 4 by treatment very well. Three species fit the model particularly poorly (Sp. 5, 9, and 16), and are also some of the species with the smallest amount of data.
(DOCX)

Table S2 Parameters solved for equation 4 fit by treatment with observed and simulated frequency for each species and treatment.
(DOCX)

Table S3 MANOVA (identity response) of all parameters fit for equation 4.
(DOCX)

Table S4 GLMM response for parameters solved for equation 4 fit by treatment.
(DOCX)

Table S5 MANOVA (identity) of characteristics of simulation.
(DOCX)

Table S6 GLMM response for characteristics of the simulated populations
(DOCX)
