# Supplementary figures and images for: Life Form and Life History Explain Variation in Population Processes in a Grassland Community Invaded by Exotic Plants and Mammals
Source: PLoS One. 2012 Aug 20;7(8):e42906. doi: 10.1371/journal.pone.0042906 (PMC3423431; doi:10.1371/journal.pone.0042906)

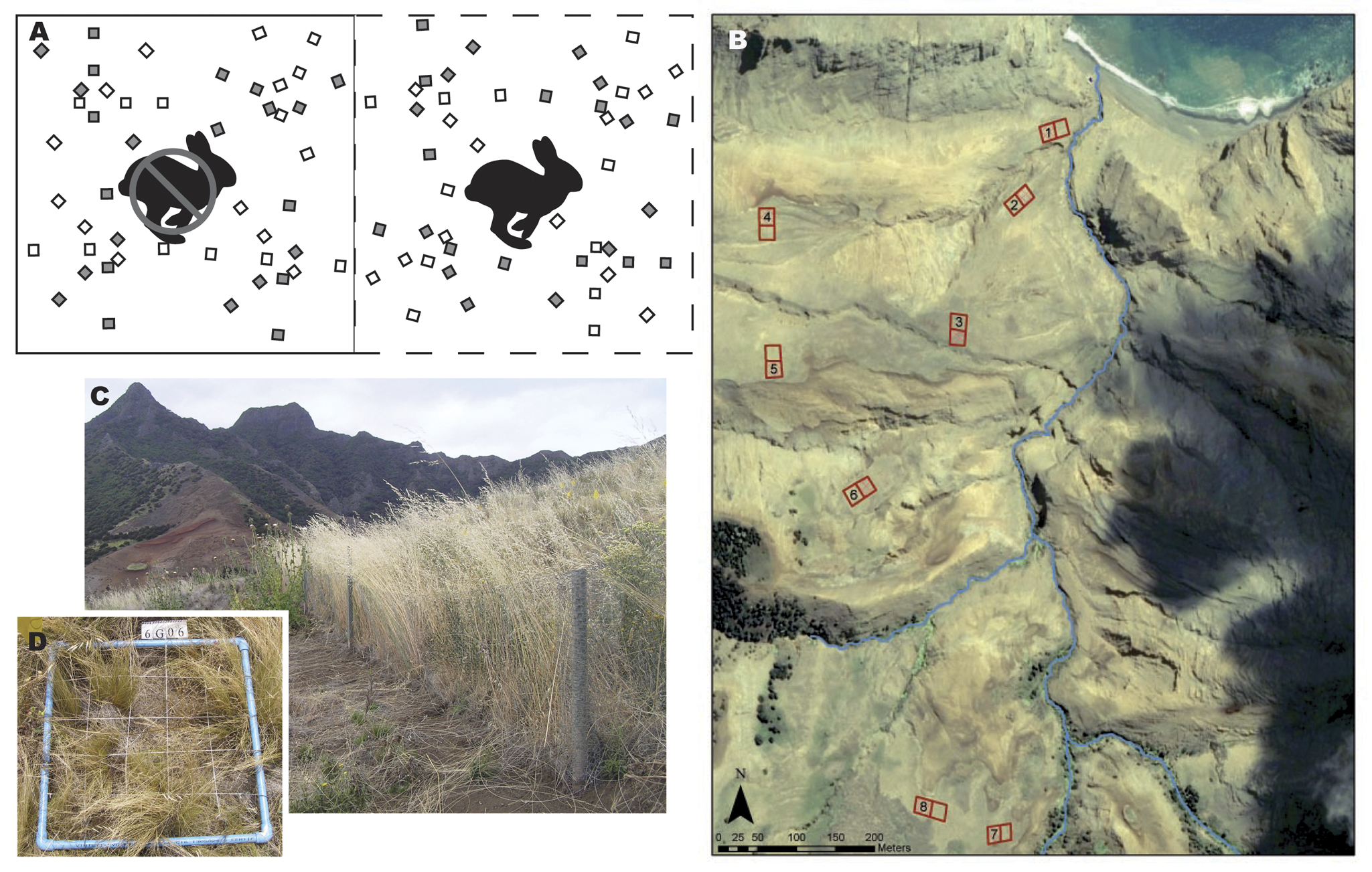

Supplement: Figure S1 — Experimental rabbit exclusion and disturbance blocks. A) Diagram of experimental blocks. Solid lines indicate fencing, dashed lines indicate fence posts but no fence. Filled squares are disturbed subplots, empty squares are undisturbed subplots. B) A map showing location of all eight blocks overlaid onto a satellite photo of Vaquería valley. The blue line is the stream. For scale, the small dark dot to the left of the stream near the beach is the cabin were researchers stay when in the valley. C) A photo of block 6 in 2005. To the left is outside of the fenced area, to the right is inside. D) This picture shows a subplot with a portable quadrat on it for collecting presence data. (TIF) [file pone.0042906.s001.tif]

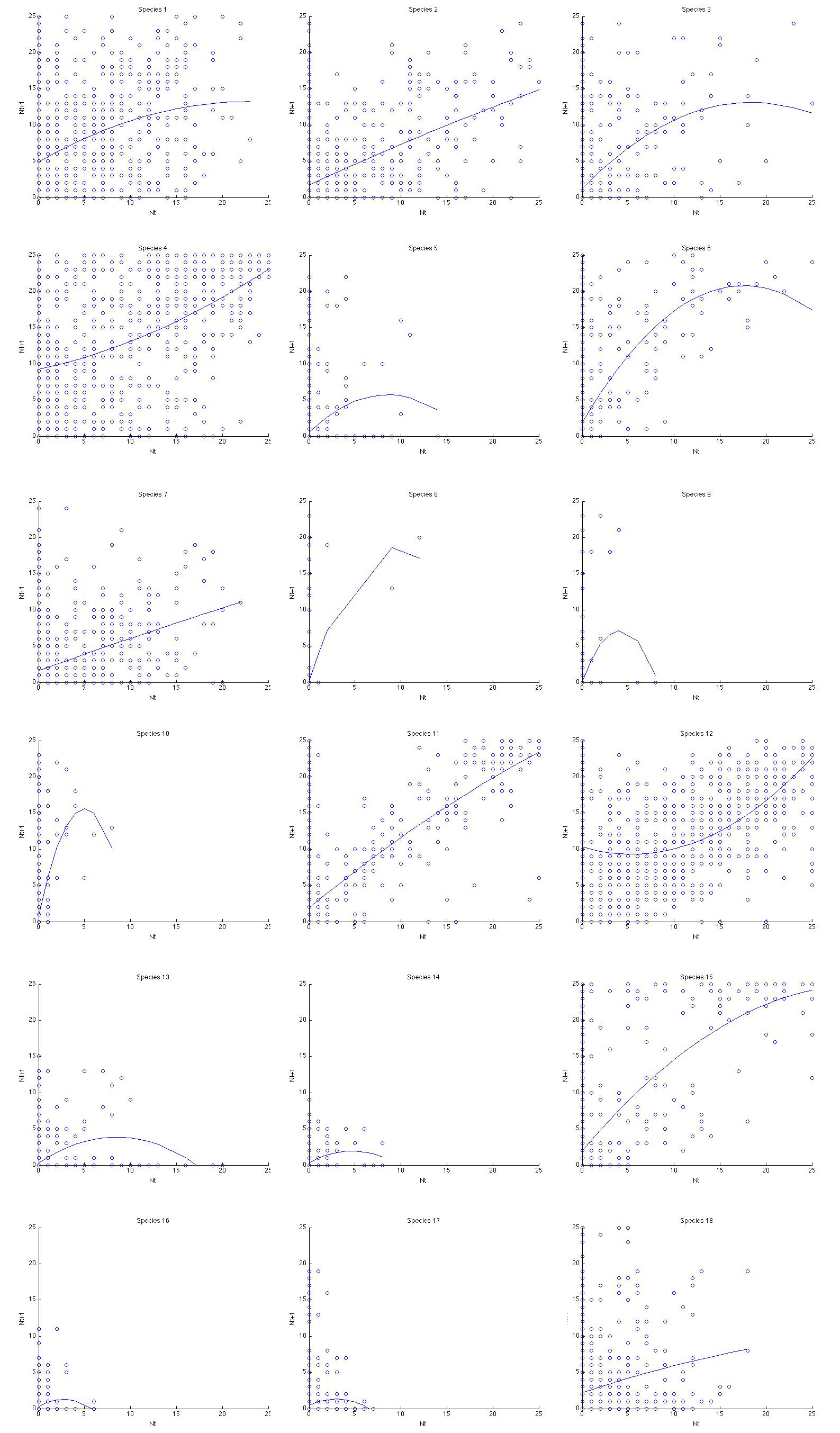

Supplement: Figure S3 — Nt versus Nt+1 for each species 2005–2007. For many of the species (3, 5, 6, 9, 10, 13, 14, 16, and 17) the curve is a textbook Ricker curve. For other species (1, 8, and 15) the curvature of the line resembles a Ricker curve that does not descend. The remaining species (2, 4, 7, 11, 12, and 18) do not resemble Ricker curves. However, some of the species that do not resemble Ricker curves fit the model exceptionally well. For example, species 11 (Juncus imbricatus) has an R2 of 0.916. (TIF) [file pone.0042906.s003.tif]

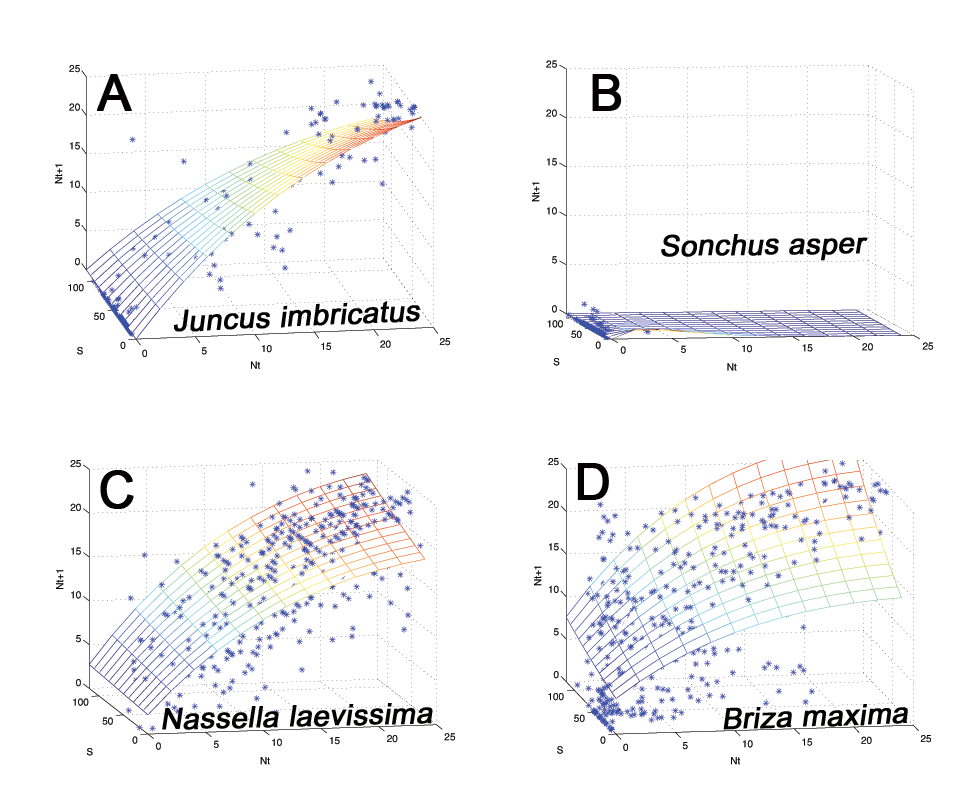

Supplement: Figure S4 — Model fit with data. The model fit for model 4 for four species in the no rabbit no disturbance treatment are shown. A) Juncus imbricatus (species 11), has the best fit with an R2 of 0.916. B) Sonchus asper (species 16) has the worst fit with an R2 of −0.040, this appears to be due to a lack of data. C) Nassella laevissima (species 12) is the dominant bunch grass and fits the model well with an R2 of 0.511. D) Briza maxima (species 4) is the dominant exotic species and fits the model well with an R2 of 0.400. (TIF) [file pone.0042906.s004.tif]

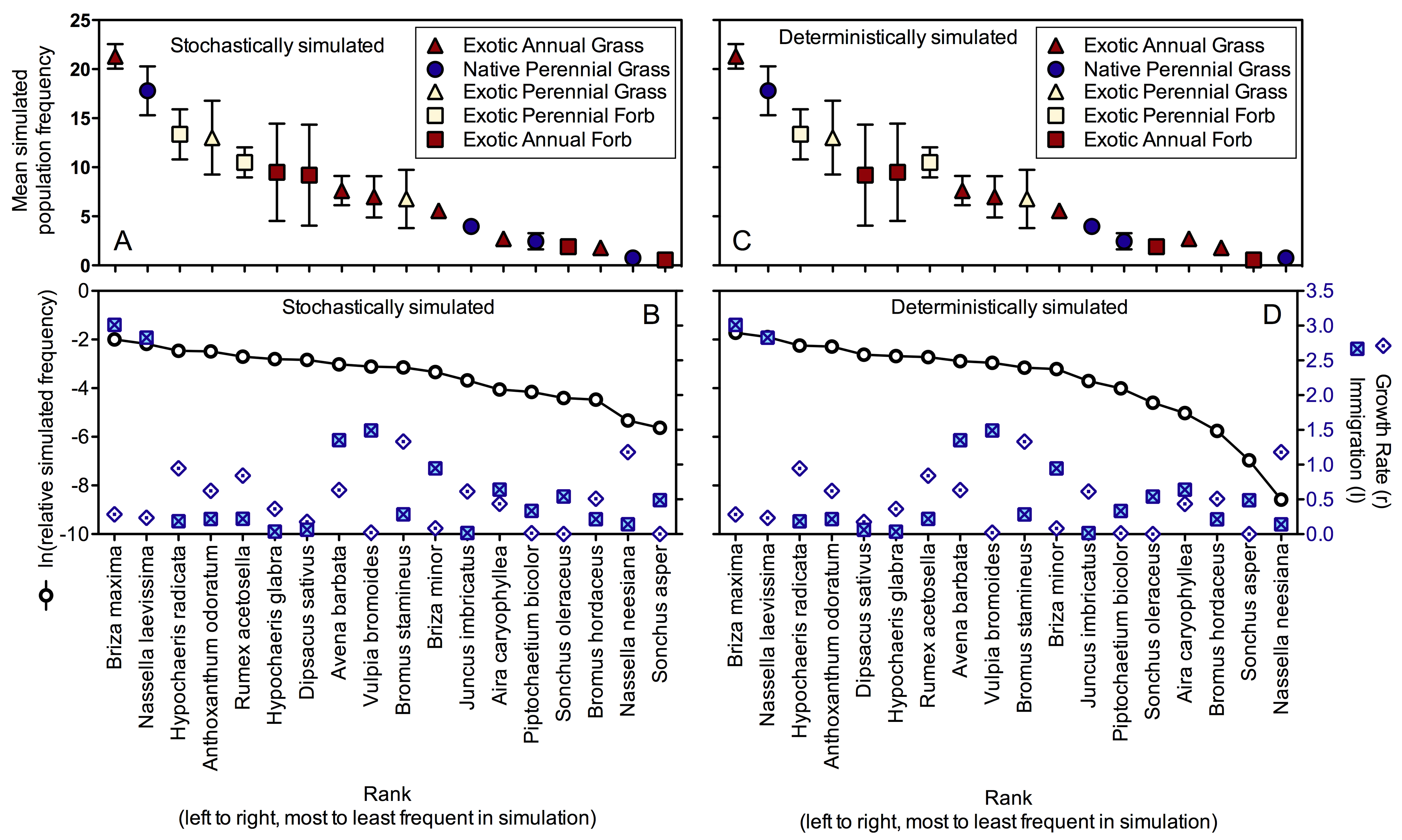

Supplement: Figure S7 — Rank-frequency, mean frequency, immigration, and growth rate of simulated data. Rank-abundance style graphs with A) mean simulated population frequency, and B) rank-frequency of stochastic model. C) mean simulated frequency, and D) rank-frequency of deterministically simulated model. Immigration (diamonds) and growth rate (squares with an x inside) are shown on the right axis. (TIF) [file pone.0042906.s007.tif]

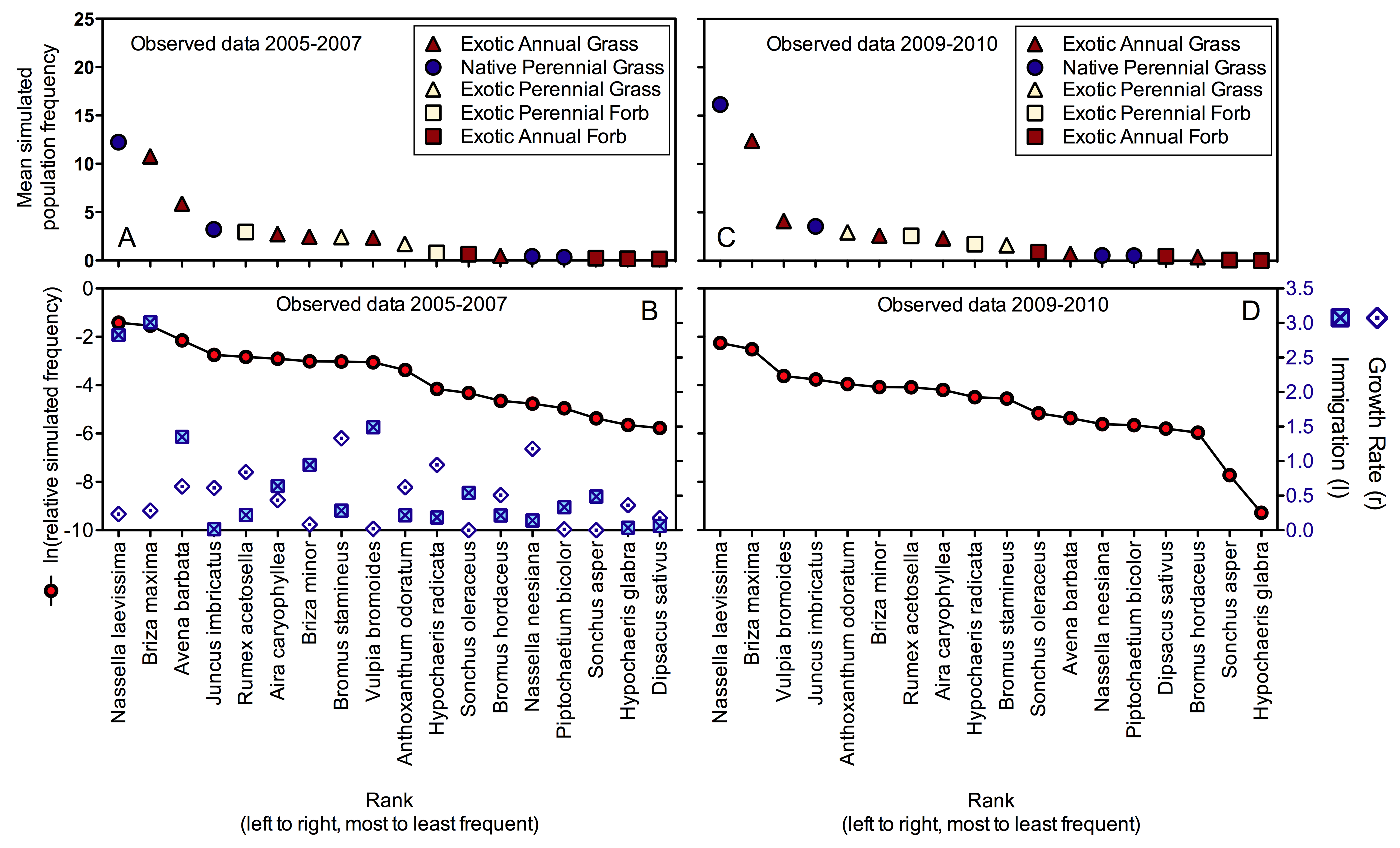

Supplement: Figure S8 — Rank-frequency, mean frequency, immigration, and growth rate of simulated data. Rank-abundance style graphs with A) mean observed population frequency 2005–2007, and B) rank-frequency of observed data 2005–2007. C) mean observed frequency 2009–2010, and D) rank-frequency of observed data 2009–2010. Immigration (diamonds) and growth rate (squares with an x inside) are shown on the right axis. (TIF) [file pone.0042906.s008.tif]
