# Supplementary material for: Life Form and Life History Explain Variation in Population Processes in a Grassland Community Invaded by Exotic Plants and Mammals
Source: PLoS One. 2012 Aug 20;7(8):e42906. doi: 10.1371/journal.pone.0042906 (PMC3423431; doi:10.1371/journal.pone.0042906)
Supplement: Table S1 — AIC and R2 fit. Columns two through nine show the AICs for each equation and treatment fit. Column ten shows the fit (numbered 1–8 in parentheses in column titles) that had the lowest AIC. The difference between the lowest AIC and the overall best fit equation, Equation 4 by Treatment (fit 8), is shown in the penultimate column. The final column shows the R2 for the fit between Eq. 4 by Treatment to the data for each species. As you can see, most of the model fits that have an AIC lower than that of Eq. 4 by Treatment still fit Eq. 4 by treatment very well. Three species fit the model particularly poorly (Sp. 5, 9, and 16), and are also some of the species with the smallest amount of data. (DOCX) [file pone.0042906.s011.docx]

| Table S1. AIC and R^2^ fit. Columns two through nine show the AICs for each equation and treatment fit. Column ten shows the fit (numbered 1-8 in parentheses in column titles) that had the lowest AIC. The difference between the lowest AIC and the overall best fit equation, Equation 4 by Treatment (fit 8), is shown in the penultimate column. The final column shows the R^2^ for the fit between Eq. 4 by Treatment to the data for each species. As you can see, most of the model fits that have an AIC lower than that of Eq. 4 by Treatment still fit Eq. 4 by treatment very well. Three species fit the model particularly poorly (Sp. 5, 9, and 16), and are also some of the species with the smallest amount of data. | | | | | | | | | | | |
| --- | --- | --- | --- | --- | --- | --- | --- | --- | --- | --- | --- |
| Species Number | Equation 1 Pooled (Fit 1) | Equation 2 Pooled (Fit 2) | Equation 3 Pooled (Fit 3) | Equation 4 Pooled (Fit 4) | Equation 1 Treatments (Fit 5) | Equation 2 Treatments (Fit 6) | Equation 3 Treatments (Fit 7) | Equation 4 Treatments (Fit 8) | Fit with the best AIC | Best AIC - Eq. 4 Treatments | R^2^ for Eq. 4 Treatments |
| 1 | 9692.64 | 9650.48 | 9643.32 | 9980.00 | 9607.39 | 9559.73 | 9552.39 | 9543.43 | 8 | 0.00 | 0.552 |
| 2 | 8348.18 | 8326.27 | 8327.96 | 8694.57 | 8167.18 | 8150.88 | 8151.63 | 8130.98 | 8 | 0.00 | 0.580 |
| 3 | 8181.93 | 8171.43 | 8165.77 | 8397.08 | 7949.13 | 7944.66 | 7946.53 | 7923.26 | 8 | 0.00 | 0.670 |
| 4 | 10348.21 | 10236.05 | 10245.23 | 10728.90 | 10375.60 | 10247.93 | 10254.25 | 10244.54 | 2 | 8.49 | 0.401 |
| 5 | 6694.23 | 6676.54 | 6677.05 | 7143.33 | 6477.54 | 6466.78 | 6463.65 | 6467.29 | 7 | 3.63 | 0.070 |
| 6 | 8870.70 | 8858.05 | 8858.36 | 9294.43 | 8725.74 | 8717.95 | 8717.24 | 8692.05 | 8 | 0.00 | 0.859 |
| 7 | 8328.02 | 8275.16 | 8277.15 | 8254.58 | 8248.56 | 8196.73 | 8207.22 | 8198.63 | 6 | 1.90 | 0.211 |
| 8 | 5287.33 | 5285.72 | 5280.05 | 5778.78 | 1802.32 | 1807.04 | 1808.49 | 1814.57 | 5 | 12.24 | 0.564 |
| 9 | 5411.09 | 5413.18 | 5409.48 | 5840.38 | 4429.13 | 4430.94 | 4393.21 | 4377.90 | 8 | 0.00 | -0.027 |
| 10 | 7398.59 | 7378.94 | 7379.63 | 7802.52 | 6888.85 | 6864.60 | 6868.34 | 6868.47 | 6 | 3.87 | 0.569 |
| 11 | 8969.95 | 8984.44 | 8975.51 | 9683.12 | 8907.79 | 8931.16 | 8923.19 | 8922.08 | 5 | 14.29 | 0.916 |
| 12 | 9628.21 | 9573.46 | 9561.25 | 10159.90 | 9506.58 | 9436.08 | 9433.28 | 9434.63 | 7 | 1.35 | 0.511 |
| 13 | 5512.63 | 5503.91 | 5479.36 | 5551.45 | 5105.51 | 5103.51 | 5089.57 | 5021.63 | 8 | 0.00 | 0.532 |
| 14 | 4577.32 | 4487.18 | 4497.42 | 4482.56 | 4527.42 | 4434.37 | 4444.76 | 4443.18 | 6 | 8.81 | 0.221 |
| 15 | 9369.08 | 9366.45 | 9369.16 | 9973.46 | 9370.62 | 9375.41 | 9370.14 | 9374.33 | 2 | 7.88 | 0.821 |
| 16 | 4420.91 | 4367.02 | 4363.25 | 4772.63 | 3485.21 | 3435.59 | 3280.70 | 3248.77 | 8 | 0.00 | -0.041 |
| 17 | 6553.11 | 6492.89 | 6428.06 | 6593.67 | 6455.06 | 6346.33 | 6325.13 | 6336.76 | 7 | 11.63 | 0.165 |
| 18 | 8721.11 | 8566.65 | 8566.33 | 8527.27 | 8497.23 | 8325.92 | 8333.04 | 8323.27 | 8 | 0.00 | 0.215 |
