# Supplementary material for: Life Form and Life History Explain Variation in Population Processes in a Grassland Community Invaded by Exotic Plants and Mammals
Source: PLoS One. 2012 Aug 20;7(8):e42906. doi: 10.1371/journal.pone.0042906 (PMC3423431; doi:10.1371/journal.pone.0042906)
Supplement: Table S2 — Parameters solved for equation 4 fit by treatment with observed and simulated frequency for each species and treatment. (DOCX) [file pone.0042906.s012.docx]

**Table S2.** Parameters solved for equation 4 fit by treatment with observed and simulated frequency for each species and treatment.

| **Species Number** | **Species** | **Native Status** | **Life-Form** | **Life-History** | **Rab-bit** | **Dist-urbance** | ***I*** | ***r*** | ***alpha*** | ***beta*** | **observed frequency** | **simulated frequency** |
| --- | --- | --- | --- | --- | --- | --- | --- | --- | --- | --- | --- | --- |
| 1 | Avena barbata | exotic | grass | annual | 0 | 0 | 0.6811 | 0.6494 | -0.0439 | -0.0012 | 5.000 | 10.049 |
| 2 | Aira caryophyllea | exotic | grass | annual | 0 | 0 | 0.3091 | 0 | -0.0031 | -0.0059 | 1.528 | 1.564 |
| 3 | Anthoxanthum odoratum | exotic | grass | perennial | 0 | 0 | 0.0661 | 1.4133 | -0.1048 | -0.0099 | 1.033 | 1.855 |
| 4 | Briza maxima | exotic | grass | annual | 0 | 0 | 3.6001 | 0.1482 | -0.0265 | 0.005 | 9.486 | 23.819 |
| 5 | Bromus hordaceus | exotic | grass | annual | 0 | 0 | 0.3069 | 0 | -0.0584 | -0.0085 | 0.328 | 0.979 |
| 6 | Bromus stamineus | exotic | grass | annual | 0 | 0 | 0.2047 | 0.8486 | -0.0382 | -0.0013 | 2.955 | 16.902 |
| 7 | Briza minor | exotic | grass | annual | 0 | 0 | 0.5547 | 0 | -0.0806 | 0.0044 | 1.399 | 7.349 |
| 8 | Dipsacus sativus | exotic | forb | annual | 0 | 0 | 0.1022 | 0.6695 | -0.02 | -0.0029 | 0.265 | 11.669 |
| 9 | Hypochaeris glabra | exotic | forb | annual | 0 | 0 | 0.0002 | 0 | -0.4002 | -0.0402 | 0.035 | 0.147 |
| 10 | Hypochaeris radicata | exotic | forb | perennial | 0 | 0 | 0.1938 | 0.9235 | -0.0727 | 0.0037 | 1.251 | 19.332 |
| 11 | Juncus imbricatus | native | grass | perennial | 0 | 0 | 0.0001 | 0.6798 | -0.0264 | -0.0034 | 3.988 | 5.323 |
| 12 | Nassella laevissima | native | grass | perennial | 0 | 0 | 1.5163 | 0.3743 | -0.0262 | 0.0018 | 14.838 | 21.853 |
| 13 | Nassella neesiana | native | grass | perennial | 0 | 0 | 0.1059 | 1.1053 | -0.0679 | -0.0277 | 0.725 | 0.936 |
| 14 | Piptochaetium bicolor | native | grass | perennial | 0 | 0 | 0.1386 | 0.0474 | -0.2649 | 0.0087 | 0.394 | 5.064 |
| 15 | Rumex acetosella | exotic | forb | perennial | 0 | 0 | -0.0003 | 0.5336 | -0.0264 | -0.0007 | 3.125 | 14.044 |
| 16 | Sonchus asper | exotic | forb | annual | 0 | 0 | 0.0002 | 0 | -0.4002 | -0.0402 | 0.049 | 0.127 |
| 17 | Sonchus oleraceus | exotic | forb | annual | 0 | 0 | 0.6682 | 0 | -0.0197 | -0.0065 | 0.721 | 1.837 |
| 18 | Vulpia bromoides | exotic | grass | annual | 0 | 0 | 1.2955 | 0 | -0.0621 | 0.006 | 2.357 | 14.036 |
| 1 | Avena barbata | exotic | grass | annual | 0 | 1 | 0.8029 | 1.1041 | -0.0605 | -0.0077 | 8.136 | 4.863 |
| 2 | Aira caryophyllea | exotic | grass | annual | 0 | 1 | 0.4721 | 0.5022 | -0.027 | -0.0096 | 2.584 | 2.185 |
| 3 | Anthoxanthum odoratum | exotic | grass | perennial | 0 | 1 | 0.2285 | 1.0752 | -0.0504 | -0.0025 | 2.600 | 12.372 |
| 4 | Briza maxima | exotic | grass | annual | 0 | 1 | 4.8377 | 0.105 | -0.0262 | 0.0036 | 11.616 | 22.244 |
| 5 | Bromus hordaceus | exotic | grass | annual | 0 | 1 | 0.0848 | 2.04 | -0.2898 | -0.0184 | 0.689 | 1.718 |
| 6 | Bromus stamineus | exotic | grass | annual | 0 | 1 | 0.4484 | 1.2773 | -0.0462 | -0.0074 | 3.590 | 4.894 |
| 7 | Briza minor | exotic | grass | annual | 0 | 1 | 0.8195 | 0 | -0.0526 | 0.0018 | 2.861 | 6.323 |
| 8 | Dipsacus sativus | exotic | forb | annual | 0 | 1 | 0.15 | 0.0415 | -0.0007 | 0.0018 | 0.346 | 25.000 |
| 9 | Hypochaeris glabra | exotic | forb | annual | 0 | 1 | 0.0002 | 0 | -0.0267 | 0.0092 | 0.190 | 24.999 |
| 10 | Hypochaeris radicata | exotic | forb | perennial | 0 | 1 | 0.1598 | 1.2676 | -0.0821 | 0.0003 | 1.104 | 15.588 |
| 11 | Juncus imbricatus | native | grass | perennial | 0 | 1 | 0.0104 | 0.6666 | -0.0264 | -0.0032 | 2.769 | 4.963 |
| 12 | Nassella laevissima | native | grass | perennial | 0 | 1 | 2.1525 | 0.1088 | -0.0263 | 0.0034 | 8.732 | 21.353 |
| 13 | Nassella neesiana | native | grass | perennial | 0 | 1 | 0.3634 | 0.1233 | 0.0213 | -0.0106 | 0.395 | 1.029 |
| 14 | Piptochaetium bicolor | native | grass | perennial | 0 | 1 | 0.3998 | 0 | -0.1214 | 0.0015 | 0.367 | 2.649 |
| 15 | Rumex acetosella | exotic | forb | perennial | 0 | 1 | 0.1333 | 0.689 | -0.0265 | -0.0019 | 3.377 | 12.979 |
| 16 | Sonchus asper | exotic | forb | annual | 0 | 1 | 0.1226 | 0 | -0.4002 | -0.0402 | 0.080 | 0.351 |
| 17 | Sonchus oleraceus | exotic | forb | annual | 0 | 1 | 0.5917 | 0 | 0.0138 | -0.0081 | 0.744 | 1.510 |
| 18 | Vulpia bromoides | exotic | grass | annual | 0 | 1 | 2.2393 | 0.0896 | -0.0372 | -0.0012 | 3.337 | 6.405 |
| 1 | Avena barbata | exotic | grass | annual | 1 | 0 | 1.2848 | 0.7811 | -0.0649 | -0.0105 | 4.354 | 4.476 |
| 2 | Aira caryophyllea | exotic | grass | annual | 1 | 0 | 0.4163 | 0.9042 | -0.0469 | -0.0136 | 2.521 | 2.696 |
| 3 | Anthoxanthum odoratum | exotic | grass | perennial | 1 | 0 | 0.2107 | 0 | -0.0442 | 0.0086 | 1.476 | 14.909 |
| 4 | Briza maxima | exotic | grass | annual | 1 | 0 | 1.2801 | 0.511 | -0.0265 | -0.0004 | 10.885 | 17.171 |
| 5 | Bromus hordaceus | exotic | grass | annual | 1 | 0 | 0.2185 | 0 | -0.0144 | -0.0028 | 0.415 | 2.271 |
| 6 | Bromus stamineus | exotic | grass | annual | 1 | 0 | 0.2665 | 0.9556 | -0.0265 | -0.0121 | 1.780 | 3.281 |
| 7 | Briza minor | exotic | grass | annual | 1 | 0 | 0.7353 | 0.2721 | -0.0251 | -0.0052 | 2.014 | 4.022 |
| 8 | Dipsacus sativus | exotic | forb | annual | 1 | 0 | 0 | 0 | -0.4 | -0.04 | 0.003 | 0.041 |
| 9 | Hypochaeris glabra | exotic | forb | annual | 1 | 0 | 0.043 | 0.7319 | 0.0053 | -0.0115 | 0.189 | 1.569 |
| 10 | Hypochaeris radicata | exotic | forb | perennial | 1 | 0 | 0.2112 | 0.6835 | -0.023 | -0.0066 | 0.368 | 5.361 |
| 11 | Juncus imbricatus | native | grass | perennial | 1 | 0 | 0.0318 | 0.8446 | -0.0264 | -0.0113 | 3.688 | 2.936 |
| 12 | Nassella laevissima | native | grass | perennial | 1 | 0 | 3.1381 | 0.2951 | -0.0262 | 0.0013 | 15.090 | 18.476 |
| 13 | Nassella neesiana | native | grass | perennial | 1 | 0 | 0.0952 | 1.5995 | -0.1311 | -0.0403 | 0.436 | 0.584 |
| 14 | Piptochaetium bicolor | native | grass | perennial | 1 | 0 | 0.3999 | 0 | -0.1866 | -0.0005 | 0.398 | 1.442 |
| 15 | Rumex acetosella | exotic | forb | perennial | 1 | 0 | 0.4264 | 0.8114 | -0.0264 | -0.0072 | 2.641 | 7.913 |
| 16 | Sonchus asper | exotic | forb | annual | 1 | 0 | 0.3998 | 0 | -0.2423 | -0.0066 | 0.314 | 0.862 |
| 17 | Sonchus oleraceus | exotic | forb | annual | 1 | 0 | 0.4792 | 0 | -0.4003 | 0.0053 | 0.590 | 1.677 |
| 18 | Vulpia bromoides | exotic | grass | annual | 1 | 0 | 0.9276 | 0 | -0.1718 | 0.0074 | 1.764 | 4.240 |
| 1 | Avena barbata | exotic | grass | annual | 1 | 1 | 2.6401 | 0 | -0.0266 | 0.0012 | 6.035 | 11.102 |
| 2 | Aira caryophyllea | exotic | grass | annual | 1 | 1 | 1.3602 | 0.3371 | -0.027 | -0.0066 | 4.348 | 4.416 |
| 3 | Anthoxanthum odoratum | exotic | grass | perennial | 1 | 1 | 0.3654 | 0 | -0.0454 | 0.0118 | 1.776 | 22.928 |
| 4 | Briza maxima | exotic | grass | annual | 1 | 1 | 2.3327 | 0.3765 | -0.0271 | 0.0029 | 11.172 | 21.974 |
| 5 | Bromus hordaceus | exotic | grass | annual | 1 | 1 | 0.2445 | 0 | -0.0386 | -0.0019 | 0.489 | 2.220 |
| 6 | Bromus stamineus | exotic | grass | annual | 1 | 1 | 0.2213 | 2.2418 | -0.0897 | -0.0306 | 1.417 | 2.009 |
| 7 | Briza minor | exotic | grass | annual | 1 | 1 | 1.682 | 0.0587 | -0.0263 | -0.0036 | 3.602 | 4.633 |
| 8 | Dipsacus sativus | exotic | forb | annual | 1 | 1 | 0 | 0 | -0.4 | -0.04 | 0.009 | 0.073 |
| 9 | Hypochaeris glabra | exotic | forb | annual | 1 | 1 | 0.107 | 0.7171 | -0.0902 | 0.0029 | 0.294 | 11.266 |
| 10 | Hypochaeris radicata | exotic | forb | perennial | 1 | 1 | 0.1803 | 0.9097 | -0.019 | -0.0062 | 0.424 | 13.138 |
| 11 | Juncus imbricatus | native | grass | perennial | 1 | 1 | 0.0274 | 0.2599 | -0.0078 | -0.0032 | 2.466 | 2.625 |
| 12 | Nassella laevissima | native | grass | perennial | 1 | 1 | 4.5009 | 0.1643 | -0.0263 | -0.0024 | 10.294 | 9.487 |
| 13 | Nassella neesiana | native | grass | perennial | 1 | 1 | 0.0002 | 1.8889 | -0.2733 | -0.0346 | 0.163 | 0.487 |
| 14 | Piptochaetium bicolor | native | grass | perennial | 1 | 1 | 0.3998 | 0 | -0.3288 | -0.0059 | 0.257 | 0.675 |
| 15 | Rumex acetosella | exotic | forb | perennial | 1 | 1 | 0.3162 | 1.3337 | -0.0483 | -0.0081 | 2.677 | 7.058 |
| 16 | Sonchus asper | exotic | forb | annual | 1 | 1 | 1.4366 | 0 | -0.2387 | -0.0221 | 0.487 | 0.904 |
| 17 | Sonchus oleraceus | exotic | forb | annual | 1 | 1 | 0.4248 | 0 | -0.3602 | 0.0081 | 0.603 | 2.626 |
| 18 | Vulpia bromoides | exotic | grass | annual | 1 | 1 | 1.5088 | 0 | -0.1144 | -0.0008 | 1.972 | 3.270 |
