# Supplementary material for: Life Form and Life History Explain Variation in Population Processes in a Grassland Community Invaded by Exotic Plants and Mammals
Source: PLoS One. 2012 Aug 20;7(8):e42906. doi: 10.1371/journal.pone.0042906 (PMC3423431; doi:10.1371/journal.pone.0042906)
Supplement: Table S3 — MANOVA (identity response) of all parameters fit for equation 4. (DOCX) [file pone.0042906.s013.docx]

| **Table S3.** MANOVA (identity response) of all parameters fit for equation 4. | | | | | |
| --- | --- | --- | --- | --- | --- |
| N=72 |  | DFE=66 |  |  |  |
|  |  |  |  |  |  |
| **Whole Model** | Value | Approx. F | NumDF | DenDF | Prob>F |
| Wilks' Lambda | 0.5268941 | 2.2367 | 20 | 209.9 | 0.0026 |
| Pillai's Trace | 0.5482219 | 2.0965 | 20 | 264 | 0.0047 |
| Hotelling-Lawley | 0.7580707 | 2.3479 | 20 | 131.34 | 0.0022 |
| Roy's Max Root | 0.4890016 | 6.4548 | 5 | 66 | <.0001 |
|  |  |  |  |  |  |
|  |  |  |  |  |  |
| Intercept | Value | Exact F | NumDF | DenDF | Prob>F |
| F Test | 4.8492451 | 76.3756 | 4 | 63 | <.0001 |
|  |  |  |  |  |  |
| **native** | Value | Exact F | NumDF | DenDF | Prob>F |
| F Test | 0.2538225 | 3.9977 | 4 | 63 | 0.0059 |
|  |  |  |  |  |  |
| **grass** | Value | Exact F | NumDF | DenDF | Prob>F |
| F Test | 0.3735412 | 5.8833 | 4 | 63 | 0.0004 |
|  |  |  |  |  |  |
| **annual** | Value | Exact F | NumDF | DenDF | Prob>F |
| F Test | 0.3688965 | 5.8101 | 4 | 63 | 0.0005 |
|  |  |  |  |  |  |
| **Rabbit** | Value | Exact F | NumDF | DenDF | Prob>F |
| F Test | 0.0534796 | 0.8423 | 4 | 63 | 0.5036 |
|  |  |  |  |  |  |
| **Disturbance** | Value | Exact F | NumDF | DenDF | Prob>F |
| F Test | 0.0222296 | 0.3501 | 4 | 63 | 0.843 |
