# Supplementary material for: Life Form and Life History Explain Variation in Population Processes in a Grassland Community Invaded by Exotic Plants and Mammals
Source: PLoS One. 2012 Aug 20;7(8):e42906. doi: 10.1371/journal.pone.0042906 (PMC3423431; doi:10.1371/journal.pone.0042906)
Supplement: Table S4 — GLMM response for parameters solved for equation 4 fit by treatment. (DOCX) [file pone.0042906.s014.docx]

| **Table S4.** GLMM response for parameters solved for equation 4 fit by treatment | | | | | |
| --- | --- | --- | --- | --- | --- |
| Immigration (*I*) |  |  |  |  |  |
| RSquare | 0.564788 |  |  |  |  |
| Source | Nparm | DF | DFDen | F Ratio | Prob > F |
| Rabbit | 1 | 1 | 52 | 1.2987 | 0.2597 |
| Disturbance | 1 | 1 | 52 | 1.7084 | 0.1969 |
| Life history | 1 | 1 | 14 | 0.02 | 0.8896 |
| Life form | 1 | 1 | 14 | 7.3123 | **0.0171** |
| Native | 1 | 1 | 14 | 1.2643 | 0.2798 |
|  |  |  |  |  |  |
|  |  |  |  |  |  |
| Growth Rate (*r*) |  |  |  |  |  |
| RSquare | 0.457933 |  |  |  |  |
| Source | Nparm | DF | DFDen | F Ratio | Prob > F |
| Rabbit | 1 | 1 | 52 | 0.0026 | 0.9595 |
| Disturbance | 1 | 1 | 52 | 0.1742 | 0.6781 |
| Life history | 1 | 1 | 14 | 13.5991 | **0.0024** |
| Life form | 1 | 1 | 14 | 0.8593 | 0.3696 |
| Native | 1 | 1 | 14 | 4.364 | 0.0554 |
|  |  |  |  |  |  |
|  |  |  |  |  |  |
| Self-regulation (α) |  |  |  |  |  |
| RSquare | 0.28417 |  |  |  |  |
| Source | Nparm | DF | DFDen | F Ratio | Prob > F |
| Rabbit | 1 | 1 | 52 | 1.2349 | 0.2716 |
| Disturbance | 1 | 1 | 52 | 0.0407 | 0.8409 |
| Life history | 1 | 1 | 14 | 0.3545 | 0.5611 |
| Life form | 1 | 1 | 14 | 0.8972 | 0.3596 |
| Native | 1 | 1 | 14 | 0.6167 | 0.4454 |
|  |  |  |  |  |  |
|  |  |  |  |  |  |
| Resistance (β) |  |  |  |  |  |
| RSquare | 0.489079 |  |  |  |  |
| Source | Nparm | DF | DFDen | F Ratio | Prob > F |
| Rabbit | 1 | 1 | 52 | 0.7232 | 0.399 |
| Disturbance | 1 | 1 | 52 | 0.1656 | 0.6857 |
| Life history | 1 | 1 | 14 | 0.4148 | 0.5299 |
| Life form | 1 | 1 | 14 | 2.0067 | 0.1785 |
| Native | 1 | 1 | 14 | 0.9497 | 0.3463 |
