# Supplementary material for: Life Form and Life History Explain Variation in Population Processes in a Grassland Community Invaded by Exotic Plants and Mammals
Source: PLoS One. 2012 Aug 20;7(8):e42906. doi: 10.1371/journal.pone.0042906 (PMC3423431; doi:10.1371/journal.pone.0042906)
Supplement: Table S5 — MANOVA (identity) of characteristics of simulation. (DOCX) [file pone.0042906.s015.docx]

| **Table S5.** MANOVA (identity) of characteristics of simulation | | | | | |
| --- | --- | --- | --- | --- | --- |
| N=72 | DFE=66 |  |  |  |  |
|  |  |  |  |  |  |
| **Whole Model** | Value | Approx. F | NumDF | DenDF | Prob>F |
| Wilks' Lambda | 0.4037238 | 3.3009 | 20 | 209.9 | <.0001 |
| Pillai's Trace | 0.7662272 | 3.1277 | 20 | 264 | <.0001 |
| Hotelling-Lawley | 1.0897973 | 3.3753 | 20 | 131.34 | <.0001 |
| Roy's Max Root | 0.547214 | 7.2232 | 5 | 66 | <.0001 |
|  |  |  |  |  |  |
| Intercept | Value | Exact F | NumDF | DenDF | Prob>F |
| F Test | 15.046345 | 236.9799 | 4 | 63 | <.0001 |
|  |  |  |  |  |  |
| **native** | Value | Exact F | NumDF | DenDF | Prob>F |
| F Test | 0.3437057 | 5.4134 | 4 | 63 | 0.0008 |
|  |  |  |  |  |  |
| **grass** | Value | Exact F | NumDF | DenDF | Prob>F |
| F Test | 0.3896267 | 6.1366 | 4 | 63 | 0.0003 |
|  |  |  |  |  |  |
| **annual** | Value | Exact F | NumDF | DenDF | Prob>F |
| F Test | 0.2914026 | 4.5896 | 4 | 63 | 0.0026 |
|  |  |  |  |  |  |
| **Rabbit** | Value | Exact F | NumDF | DenDF | Prob>F |
| F Test | 0.1807913 | 2.8475 | 4 | 63 | 0.031 |
|  |  |  |  |  |  |
| **Disturbance** | Value | Exact F | NumDF | DenDF | Prob>F |
| F Test | 0.0519708 | 0.8185 | 4 | 63 | 0.5182 |
