# Supplementary material for: Life Form and Life History Explain Variation in Population Processes in a Grassland Community Invaded by Exotic Plants and Mammals
Source: PLoS One. 2012 Aug 20;7(8):e42906. doi: 10.1371/journal.pone.0042906 (PMC3423431; doi:10.1371/journal.pone.0042906)
Supplement: Table S6 — GLMM response for characteristics of the simulated populations (DOCX) [file pone.0042906.s016.docx]

**Table S6.** GLMM response for characteristics of the simulated populations

| Coefficient of Variation | |  |  |  |  |
| --- | --- | --- | --- | --- | --- |
| RSquare | 0.556172 |  |  |  |  |
| Source | Nparm | DF | DFDen | F Ratio | Prob > F |
| Rabbit | 1 | 1 | 52 | 1.1147 | 0.296 |
| Disturbance | 1 | 1 | 52 | 0.0364 | 0.8494 |
| Life history | 1 | 1 | 14 | 4.8172 | **0.0455** |
| Life form | 1 | 1 | 14 | 0.6738 | 0.4255 |
| Native | 1 | 1 | 14 | 2.9597 | 0.1074 |
|  |  |  |  |  |  |
|  |  |  |  |  |  |
| Number of times extinct | |  |  |  |  |
| RSquare | 0.564155 |  |  |  |  |
| Source | Nparm | DF | DFDen | F Ratio | Prob > F |
| Rabbit | 1 | 1 | 52 | 1.8581 | 0.1787 |
| Disturbance | 1 | 1 | 52 | 0.0115 | 0.915 |
| Life history | 1 | 1 | 14 | 5.3815 | **0.036** |
| Life form | 1 | 1 | 14 | 0.42 | 0.5274 |
| Native | 1 | 1 | 14 | 2.7691 | 0.1183 |
|  |  |  |  |  |  |
|  |  |  |  |  |  |
| Mean frequency | |  |  |  |  |
| RSquare | 0.544167 |  |  |  |  |
| Source | Nparm | DF | DFDen | F Ratio | Prob > F |
| Rabbit | 1 | 1 | 52 | 3.0544 | 0.0864 |
| Disturbance | 1 | 1 | 52 | 0.9242 | 0.3408 |
| Life history | 1 | 1 | 14 | 6.0636 | **0.0274** |
| Life form | 1 | 1 | 14 | 4.4493 | 0.0534 |
| Native | 1 | 1 | 14 | 6.1094 | **0.0269** |
|  |  |  |  |  |  |
|  |  |  |  |  |  |
| Highest frequency reached | | |  |  |  |
| RSquare | 0.733384 |  |  |  |  |
| Source | Nparm | DF | DFDen | F Ratio | Prob > F |
| Rabbit | 1 | 1 | 52 | 3.7408 | 0.0586 |
| Disturbance | 1 | 1 | 52 | 2.9554 | 0.0915 |
| Life history | 1 | 1 | 14 | 4.1787 | 0.0602 |
| Life form | 1 | 1 | 14 | 3.3117 | 0.0902 |
| Native | 1 | 1 | 14 | 6.7351 | **0.0212** |
